# ‘Going private’: a qualitative comparison of medical specialists’ job satisfaction in the public and private sectors of South Africa

**DOI:** 10.1186/1478-4491-11-1

**Published:** 2013-01-03

**Authors:** John Ashmore

**Affiliations:** 1 Health Economics Unit, School of Public Health and Family Medicine, Falmouth Annex, Medical Campus, University of Cape Town, Observatory 7925, Cape Town, South Africa

**Keywords:** Job satisfaction, Retention, Private sector, Motivation

## Abstract

**Background:**

There is a highly inequitable distribution of health workers between public and private sectors in South Africa, partly due to within-country migration trends. This article elaborates what South African medical specialists find satisfying about working in the public and private sectors, at present, and how to better incentivize retention in the public sector.

**Methods:**

Seventy-four qualitative interviews were conducted - among specialists and key informants - based in one public and one private urban hospital in South Africa. Interviews were coded to determine common job satisfaction factors, both financial and non-financial in nature. This served as background to a broader study on the impacts of specialist ‘dual practice’, that is, moonlighting. All qualitative specialist respondents were engaged in dual practice, generally working in both public and private sectors. Respondents were thus able to compare what was satisfying about these sectors, having experience of both.

**Results:**

Results demonstrate that although there are strong financial incentives for specialists to migrate from the public to the private sector, public work can be attractive in some ways. For example, the public hospital sector generally provides more of a team environment, more academic opportunities, and greater opportunities to feel ‘needed’ and ‘relevant’. However, public specialists suffer under poor resource availability, lack of trust for the Department of Health, and poor perceived career opportunities. These non-financial issues of public sector dissatisfaction appeared just as important, if not more important, than wage disparities.

**Conclusions:**

The results are useful for understanding both what brings specialists to migrate to the private sector, and what keeps some working in the public sector. Policy recommendations center around boosting public sector resources and building trust of the public sector through including health workers more in decision-making, inter alia. These interventions may be more cost-effective for retention than wage increases, and imply that it is not necessarily just a matter of putting more money into the public sector to increase retention.

## Background

The inequitable distribution of health workers between public and private, rural and urban, primary and tertiary, and poor and rich settings is an important determinant of countries’ inabilities to meet the Millennium Development Goals, and to reap the rewards from scaled-up investments in essential health services such as HIV care and maternal services [1-3]. In Africa, the situation of inequitable distribution of health workers has been termed ‘critical’. The continent houses 24% of the global disease burden, including 68% of all people living with HIV [4]; yet has only 3% of the world’s health workers to tackle these problems [1].

South Africa has a relatively plentiful supply of health workers, with over four doctors, nurses, and midwives per 1,000 people, according to the World Health Organization’s (WHO) *Global Atlas*. This falls above the ‘critical’ benchmark of 2.5 health workers per 1,000 people, as defined by the Joint Learning Initiative in 2004 [5], and above most other African nations. This reflects South Africa’s status as a middle-income country with a relatively advanced medical infrastructure [6].

However, South Africa’s internal or within-country distribution of health workers is highly inequitable. The Western Cape, a rich, urbanized province in the country, has over triple the number of doctors per capita than four of the most rural provinces. Limpopo Province has only one doctor per 5,000 people, according to the Health Professions Council of South Africa in 2010.

The private sector, meanwhile, employs around 28% of all nurses, 46% of GPs, and 56% of medical specialists [7]. This is to serve the wealthiest 15% of the population with private health insurance [8]. Since all South African doctors train in the public sector, and many start their careers in rural internships, this implies health workers are migrating away from areas of highest healthcare need [9].

The situation significantly undermines South Africa’s ability to effectively tackle its public health crises, including its HIV and TB epidemics. According to the Actuarial Society of South Africa’s 2008 demographic model, almost 11% of all South Africans are HIV positive. Most recently, WHO estimated 795 TB cases per 100,000.

Retaining health workers where they are needed within South Africa is thus an important policy imperative [10]. This may be true now more than ever, given the government’s renewed commitment to provide HIV treatment after a period of institutionalized HIV ‘denialism’ [11]. There are also ongoing plans to ramp up public health services in general, in preparation for a National Health Insurance (NHI), that is, a system of universal health coverage [12,13]. Success of these ventures depends on greater health worker availability for under-served populations [14].

This article focuses on how to better retain medical specialists in the public sector of South Africa. Specialists, also known as consultants, are critical for the provision of tertiary-level services, and in training and supervising the next generation of doctors. They are also one of the most inequitably distributed of all health worker cadres between public and private sectors (see above). In order to assess how to better retain public specialists, the article explores the ways the public, relative to private, sector provides ‘job satisfaction’ to South African specialists.

The concept of job satisfaction is focused upon here since it is an important conceptual [15,16] and empirical [17-19] determinant of retention, along with other determinants including organizational loyalty [20,21]. Job satisfaction originates in the organizational psychology literature, but has been adopted by some researchers in the field of human resources for health [17,22,23]. The concept is defined by Locke as ‘a pleasurable or positive emotional state resulting from the appraisal of one’s job or job experiences’ [16]. Essentially, job satisfaction is critical to retention since if an individual is satisfied in their job, they are more likely to stay in it, that is, be retained. Conversely, if an alternative job provides higher satisfaction, an individual is more likely to leave [21,24]. This conceptualization of job satisfaction is much like that of ‘utility’, which economists similarly use to understand retention/migration behavior.

As noted by Spector [15], job satisfaction is commonly understood as determined by wages [25], by the ‘work context’, including working conditions; by the social work environment; and by enjoyment of the work itself. Job satisfaction implies workers can be considered what Ben-nur & Putterman [26] call ‘other-regarding’ and ‘process-regarding’, implying they are motivated partly by concern for others, and ethical concerns, as well as individualism. This is important since altruism and ethics explicitly form part of health workers’ professional values [27,28]. The concept of job satisfaction thus seems a well-developed and realistic way to understand retention behavior, though other studies may also use the concept of utility in a similar way.

Job satisfaction can be conceptualized in a number of different ways [29], but in this article, as in a number of strands in the literature [16,24,30], it is assumed to result from the fulfillment of individuals’ needs, as well as their values and expectations. Note that needs may be universal, as with physiological survival needs [31], whereas values and expectations are at least partly determined by organizational culture, and personal history and individual preferences [32]. In all cases, the fulfillment of needs, values, and expectations is assumed intrinsically satisfying.

Needs, values, and expectations are hierarchical, with some taking precedence over others [16,31]. For instance, basic survival needs are likely to be most important for individuals to fulfill, generally through income, as stressed by Maslow in his 1954 theory of a hierarchy of needs [31]. Once such needs are fulfilled, however, other concerns come to the fore, such as the need for recognition and praise (a social environment factor), or the value placed on mentally stimulating work (a ‘work itself’ factor). This implies, somewhat counter-intuitively, that the more individuals earn, the less prominent income becomes in decisions to stay in particular jobs [33,34]. As such, specialists, who earn well compared with other health workers, may be expected to be at least partly, if not primarily, satisfied by non-financial factors. Economists term this as a result of decreasing marginal returns to income.

Previous empirical studies have found that although doctors may have relatively few unfulfilled financial needs, their expectations of income can be high [35,36]. Thus income may be important to specialist’s career decisions in spite of earning relatively high wages to begin with. Doctors have also been found (dis)satisfied empirically, to a significant degree, by non-financial factors. These factors include physical working conditions such as the state of hospitals [23], how demanding patients are [37], degree of working autonomy [23,36], and over-working or ‘burnout’ [38]. It thus seems reasonable to expect a variety of financial and non-financial factors affecting doctors’ job satisfaction.

In South Africa, public doctors have been found dissatisfied by pay, as well as non-financial factors including: ‘lack of equipment, drugs… unsupportive management systems… [and] lack of a career structure’, especially in rural areas [39-41]. Much has been made in the media, in addition, of poor public working conditions such as dirty hospitals, lack of beds, and regular power cuts [42,43]. However, no detailed case studies have been undertaken to date of South African specialists’ job satisfaction, despite high rates of attrition. This article aims to address this gap, highlighting both reasons for specialists staying in the public sector, and reasons for migrating to the private sector.

## Methods

The qualitative evaluation of medical specialists’ job satisfaction reported on here was performed as background to a broader study on the impacts of, and motivations for, ‘dual practice’. Dual practice is also known as moonlighting, and in low- and middle-income countries such as South Africa, generally involves health workers working in both public and private sectors in tandem [44-46]. Dual practice specialists interviewed for the study provided significant and unique insight into the attractiveness of the public, relative to the private, sector, having experience of working in both.

Sampling was limited to South African dual practice doctors working in urban, hospital settings, to keep the research manageable. This excluded general practitioners (GPs) and rural doctors from the sample, but included specialists and ‘medical officers’ (MOs). Qualitative sampling was ‘purposively’ conducted, aiming for a heterogeneous sample of urban hospital doctors (including specialists) who undertook dual practice, to arrive at theoretical saturation as quickly as possible [47]. Specialists were further focused upon in analysis, as in the findings of this article, since they comprised 82% of doctor respondents interviewed (*n*=23/28). This appeared to reflect a greater likelihood of undertaking dual practice among specialists, relative to other doctors, due to higher private financial incentives, inter alia [48].

Interviews were conducted in one large public, and one private, hospital - anonymized as H1 and P1, respectively. The hospitals were located in a major city of South Africa, also anonymized to protect respondent identities. Seventy-four qualitative interviews were carried out in total: 23 among key informants, including policymakers and managers; and 51 among 28 dual practice doctors. Dual practice doctors were interviewed twice, where available for follow-up, to build trust and pursue hunches [49]. Snowball and maximum variability sampling techniques were used, the former of which is a practical means of building a respondent base, and the latter of which aims for heterogeneity in the sample.

Interviews were pursued in 6 hospital departments, and respondent heterogeneity was achieved across characteristics of age (29 to 63 years), gender (36% women), type of multiple job arrangement held (public-to-private, private-to-public, and other dual practice forms [45]), and to some degree, ethnicity (note, specialists were all white bar 3, but this appeared to reflect the demographic profile of dual practice specialists in the field sites, as documentation acquired from a key informant later confirmed). Again, it is important to stress that all doctors interviewed undertook dual practice in one form or another, though dual practice between the public and private, for-profit sector was by far the most common (*n*=25/28).

The interviewer asked free-attitude questions about dual practice and work histories first (including, ‘tell me about the history of your working life, starting from when you qualified as a doctor. I’m particularly interested in reasons for entering and leaving different jobs’). This open approach provided context on job satisfaction and reasons for retention and migration over time. It also helped avoid ‘leading’ individuals early on in their responses [50]. Respondents were prompted on reasons for staying in or leaving the public sector, and on what they got out of working in each sector. Increasingly structured questions were later asked on emerging themes, for further clarifications. After free-attitude questions, a ‘semi-structured’, more engaging and affirming, conversational interview technique was used, to encourage respondents’ openness and buy-in while elaborating on previous answers [51]. Interviews were recorded and transcribed, to retain ‘thick’ context in the data [49].

Ethical clearance for fieldwork was obtained in October 2009, for 1 year, from the University of Cape Town Health Sciences Research Ethics Committee (Reference 446/2009). The Committee approved the use of verbal consent for interviews, to protect respondents’ identities.

## Results

As noted above, interviews focused partly upon what dual practice specialists found comparatively satisfying about working in both public and private sectors. These determinants of job (dis)satisfaction were coded into the following four areas, in line with the categorizations of Spector [15] in his review of the literature:

1. **Rewards:** that is, financial incentives and benefits

2. **Work context:** non-financial incentives including those in the physical work environment and relating to working conditions

3. **Social work environment:** non-financial incentives relating to all relationships in the work environment, including with colleagues, managers, patients, and hospital and organizational management

4. **The work itself:** regarding satisfaction with the actual work undertaken for the job

Results from interviews with dual practice specialists will now be explored in each of these areas, in turn, before tabulating and summarizing the results together.

### Rewards in the public *versus* private sector

As expected, financial rewards in the private sector were much higher than in the public sector. Some specialists interviewed appeared to value high financial rewards more than others, and thus felt more desire to work in the private sector. In general, however, respondents working in public and private sectors in tandem (constituting the vast majority of participants in the study) seemed to value financial rewards considerably, since this was generally part of their reason for undertaking at least some private work. Conversely, respondents noted that if all they cared about was income, they would only work in the private sector, rather than in dual practice arrangements.

Wage differentials of up to six times were noted in the private, relative to the public, sector, depending partly on hospital department and how much specialist training individuals had undertaken. Private sector neurosurgery was said to pay much more than neurology, for instance, due to high private demand for surgical skills. Overall, wages appeared to represent a significant financial incentive to work in or migrate to the private sector.

The public sector appeared to hold some financial advantages, however. These included a state pension, paid holidays, and less potentially costly medicolegal risk than in the private sector. The latter was said to be due to specialists not being as individually responsible for patients in public, and due to lower probability of being sued. In H1, a public academic hospital, it was also possible to take paid sabbatical leave, and make free use of the research and other academic facilities. Note that in South Africa, the public sector is where the vast majority of academic teaching and research takes place.

There was also greater stability of income in the public sector. One H1 senior manager noted this could be satisfying, though potentially conducive to poor quality service provision:

"… the public sector is rock solid, so you basically have to do something bad to get fired. So there is a high degree of certainty in your job, and whether you do it well or whether you don’t do it so well you’re still going to get paid your salary. (H1 Manager 3)"

Moving into the private sector, meanwhile, was noted to involve potentially high sunk costs, including through purchasing one’s own equipment. This made it potentially expensive to migrate into the private sector. There was also no guarantee of a regular supply of private patients for specialists until private GP referral networks were established, which could take several years.

As such, the private sector did seem to pay more than the public sector in general, but the financial attractiveness of ‘going private’ was not necessarily as great as it might at first appear for everyone, due to higher job stability and good benefits in the public sector, as well as migration costs associated with establishing a private practice.

### Work context

In terms of the work context, specialists in the private sector were often noted to have to ‘sell availability’, that is, be ‘on the end of the phone’ whenever required by a patient. This was primarily since private doctors were often solely responsible for patients, in a legal sense, and because private patients tend to have more options of where to go. Having others working under you and around you in the public sector, as in H1, was thus highly valued, since it meant more predictable working hours and less ‘selling availability’.

The quote below shows a private specialist who described public work arrangements as a ‘shield’ in this respect, showing the value he placed in the protective aspects of being part of a team:

"In public you’re shielded by an intern and you’re shielded by a registrar. In private practice you don’t have that actually so you work a lot harder, so if there’s a problem with your patients late at night or after hours, you’re it, you have to resolve that problem; whereas in the government sector there are other people to resolve those problems. (P1 DP1, Interview 1)"

Unpredictable working hours were seen as a particular barrier for female specialists with children entering private practice, who were more likely to have child care responsibilities. When a specialist who was a single mother was asked why she chose to work in the public sector, one area she highlighted was that, ‘I can plan my life’.

On the other hand, the public sector was noted to have fewer resources and less equipment and drugs available, factors which hindered the ability to do one’s job as desired, often considered frustrating. Specific examples were of a screening machine that kept breaking, difficulty in obtaining scans, lack of theater time for certain elective operations, and inability to ‘teach properly’ in academic institutions:

"…you are in a teaching facility. I mean you would love to have all the modern things like the books the overseas people are talking about and you would love to impart that knowledge onto your students. But we don’t have the equipment, I mean we have but you will find that they are outdated. So at least the other advantage of being in the private sector [is] you get to see what’s current and what’s currently in use as well, which we don’t have on the other side. (H1 DP14, Interview 1)"

These issues clearly related to public sector resource constraints, also reflected among managers in consistent reminders of ‘budget constraints’, cuts in ‘theater time’, and talk of ‘political in-fighting’ among departments. Many working in the public sector seemed highly resilient in spite of such imperfect conditions, but found the interview a safe space to open up about frustration over lack of resources:

"Okay you just go and look at the lavatories, especially in the public areas… That’s the consumer, but you know there are ways you can deal with that, and one of the ways to deal is that you have some sort of attendant, and constant cleaning of the lavatories. I mean a lot of patients come to me and… refuse to go to the lavatory because they say it’s so filthy… And that makes one feel very ashamed… Telephones get stolen… bed linen gets stolen, and you’re working in that environment… where there isn’t a blanket to put on the patient, there isn’t a pillow for her head and it’s because things have been nicked. So and all of that you know is difficult. (H1 DP15, Interview 1)"

Lack of public sector staff, relative to the private sector, was another resource issue that caused public sector dissatisfaction. For instance, there was near universal condemnation of the low level of administrative and porter staff in H1. One respondent mentioned that lack of admin staff was a key frustration their department had identified in a recent meeting, and another noted how the burden can fall on doctors, as a result, whose specialist skills thus become under-utilized.

"…within every department [in H1] there are the obvious managerial requirements that some people take up. So somebody might do the roster allocation, somebody might do the leave allocation, somebody might do the budgeting, all that kind of stuff within any department. And that is left mostly to the members of the department to do, even though we have very little training or no training whatsoever in management. People just have to assume… those kind of roles in the department in South Africa. (H1 DP3, Interview 1)"

Lack of doctors themselves also caused dissatisfaction, implying there is a damaging cycle where retention is a problem, since lack of retention may encourage others to leave. The following two extracts show specific effects of lack of doctors, in terms of others having to pick up the work and feeling they cannot take leave as a result:

"I mean… in our department… to retain people is quite difficult, people work for a year or two then they go to private or they go off somewhere else. And for those posts to be filled again, it takes a lot of time… and in between people are frustrated. (H1 DP14, Interview 1)"

"…if you feel you can’t go away because there aren’t people to cover your work then it creates tension in your ability to care for people. So resources around you do matter… The deficit falls on you to work hard. (H1 DP18, Interview 2)"

Resource constraints were hardly mentioned, meanwhile, in discourse surrounding the private sector. A number of respondents described the private environment, in this regard, simply with the adjective ‘nice’:

"… it’s extremely nice being in a place where things work. If I want a new drug or I want a new instrument or something - it’s there in private. You just have to ask for it. And, you know, motivate for it, it’s not [a] free for all. But still, it’s there… (H1 DP1, Interview 1)"

There was also dissatisfaction expressed in the public sector with the sense of career progression. It was repeatedly noted how once a senior specialist in the public sector, it is easy to become ‘stuck’, for example, since there are few chief or principal specialist jobs available. There was also a definite sense that the private sector presented opportunities for more recognition of one’s experience and seniority, and thus a sense of career progression, if only through higher prestige and, relatedly, higher wages.

"…when you go into a job you need something that’s got a career path, and there aren’t career paths [in public]. There’s a few, a small little cadre at the top, a small group of people who get to principal or chief specialist, and the rest of the people can spend their entire career as a senior specialist no matter how brilliant they are and much of a contribution they make. (H1 DP15, Interview 1)"

This, in turn, seemed partly connected to unwillingness among respondents to take specialist positions in less prestigious hospitals than H1, where higher-level jobs were said to be easier to come by.

In sum, public resource constraints, attrition, and perceived lack of career progression tended to hinder satisfaction in the work context. This counter-balanced, and possibly outweighed, the positive factors of greater work predictability and ‘shielding’ from over-burdensome patient responsibility.

### Social work environment

Respondents in H1 often noted more interaction with other doctors than in P1 due to more structured teamwork (see above). Interactions thus seemed more satisfying on a social and professional level, for many, in terms of ‘collegiality’ and ‘camaraderie’ in the public sector. One young public sector-based specialist noted ‘this is how medicine should be’, after pointing to the regular interactions observed with colleagues in H1 corridors and on ward rounds. Respondents often contrasted this collegial atmosphere with the private sector, which was seen as more ‘lonely’ and ‘competitive’:

"…it’s very stimulating to work in a collegial and academic environment where you’re going to, you know, X-ray meetings and you’re on ward rounds, with consultants that are giving their different inputs; and I certainly don’t feel that in the private sector … you grow in that way because you’re on your own, you make your decision, there’s no one to question you. It’s very difficult to get other people to come and see a patient if they’re not being paid to do it… (H1 DP10, Interview 2)"

Yet there were issues of tense social relations between specialists and other hospital workers in the public sector:

"There’s a difficulty in terms of the nursing staff and I don’t think, I think when I was a registrar it was better. I think the staff were trained differently, they were trained in general nursing and then midwifery so the midwives were midwives instead of doing 3 months or whatever it is in midwifery and a general training, so they’re less competent… the doctors are picking up a lot of duties which the nurses should do automatically and they don’t. Which makes it far less satisfying for the doctor, and far more stressful because… you can’t trust the instructions are definitely going to be carried out. (H1 DP15, Interview 1)"

The reason for poor relations between specialists and nurses seemed partly due to hostile power dynamics, particularly when fatigue sets in and tempers become frayed, as explained by one respondent. Whatever the reasons, in H1 at least, relations between different health providers (rather than between doctors) were generally perceived as much better in the private sector. A private-based specialist described P1, in this respect, as the ‘most civilized place you can imagine’, where ‘everyone is polite’, as opposed to government service where there is ‘no politeness… it’s mind twisting!’

In terms of management, specialists in H1 generally seemed happy with their Heads of Department (HODs), who are specialists’ managers. In private hospitals, meanwhile, specialists are officially self-employed, and as such generally are not ‘managed’ in the same way, if at all. This was felt advantageous to private specialists in terms of having more autonomy (see below), but H1 doctors often felt supported by their HODs, which was also valued. One public-based specialist described her HOD as a ‘sympathetic’ figure who had arranged a work schedule that allowed her to spend more time with her baby.

Having supportive managers in the public sector, where this is the case, may thus represent an incentive to stay there, since effective management no doubt adds another pillar of support to specialists. Managers can also provide recognition for good performance, which everyone appeared to appreci-ate. However, one private-based respondent seemed highly embittered by the management of her old department in H1. She noted ‘promises are lip service’, and that she had been let down repeatedly by their ‘disrespect’, alluding to what she saw as inflexible, sexist and ‘exploitative’ policies, such as employing young specialists in more junior, medical officer positions. This, conversely, was part of her reason for leaving full-time public work.

However, positive sentiments did not generally extend to higher-level hospital management, the Provincial ‘administration’, or the broader Department of Health (DoH). There was very little trust mentioned of these powers, clear in discourse surrounding budget allocations, complaints procedures, and other administrative issues, including dual practice policy itself. For example:

"…you feel that you’re being hamstrung at every turn by the state in what you’re trying to do. They don’t make an effort to find out what’s required by people who are actually… doing the job. And this is across the board. That is certainly how it feels… (H1 DP13, Interview 1)"

"I don’t think… [the administration] quite realize the human resources they have available to them. I think sometimes they don’t actually realize they’re working with professionals, and they don’t treat us as such. In many other organizations, people with our skills and experience would be very highly valued and perceived as such. But you know here we don’t get perceived or treated like that at all. And [dual practice] regulations are just another way of devaluing us. (H1 DP17, Interview 1)"

The above respondents were noticeably embittered towards state and hospital management, which seemed almost universal. A number of respondents mentioned they felt they were treated like ‘children’ in the case of dual practice policy rules (known as Remunerated Work Outside the Public Service (RWOPS) rules), which require public-based specialists to make detailed work plans to show how they will maintain public duties while performing additional private work. There were also misperceptions about RWOPS rules which turned out, on further inspection, not to be true; such as that RWOPS requires specialists to perform all private work in the evening (confirmed to be untrue in RWOPS policy documents and discussion with provincial DoH policymakers).

These tensions over specific policy issues seemed symbolic of deeper problems, since the reactions were very extreme, and sometimes even based on false information. Rather, the tensions seemed to have historical roots, relating for instance to the distrust of the health system built among staff during the Mbeki government’s HIV denialism, as well as amid historically stagnant public sector funding during prolonged periods of economic growth [52]. Such issues may have culminated in a feeling that high-level management was not trustworthy, which manifested in relatively extreme frustration with relatively micro-level issues.

In the case of patient interactions, meanwhile, most seemed fairly happy. For those who felt there were issues with their relationships with patients, however, it was clear that some doctors had relatively legitimate issues, while others’ issues were underscored by racist or classist assumptions.

In terms of what might be considered ‘legitimate issues’ with patients, it was noted that public sector patients tend to be less compliant in following instructions, which can be frustrating; while private patients can be overly ‘demanding’. Both issues relate at least partly to the demand-driven nature of private medicine, and the level of education of patients, which tends to be lower in the public sector.

"[in private]… you need to satisfy the patient above all, which takes a lot out of you. You have to bend over backwards rather than just doing your job… just doing what is appropriate. (H1 DP1, Interview 2)"

"It’s also very different in private practice where patients are far more demanding, you know in the state practice patients have to just accept what you dish out to them, it’s not the same in private practice. Patients come here with an idea [of] what they want, and if they don’t get it from you they’re going to go to someone else. (P1 DP1, Interview 1)"

Racism and classism were not often completely overt, but were apparent for instance among white or Colored respondents describing primarily black public patients as a ‘clientele of gangsters’, or noting preference in treating patients who ‘can actually speak Afrikaans or English’ - a categorization which excludes only black people. One respondent went as far as describing public patients as ‘animals’:

"…you know it’s like treating animals. If I wanted to be a vet then I would have been a vet, but I didn’t want to be a vet. (P1 DP5, Interview 1)"

These issues were rarely brought up, however, and those who worked in the public sector generally seemed just as happy with their patients as private doctors, probably because those with major issues with public patients may have self-selected, that is, opted to move, into private-only work (as with the last respondent above).

In addition, the amount of specialist-patient contact seemed very different between public and private sectors. The public sector, due to ‘shielding’ of specialists by registrars and interns, generally involved less direct and regular patient interactions than the private sector, where patient contact was sometimes seen as ‘overwhelming’. While some valued regular patient interaction more than others, there was generally a feeling that not having shielding was ‘stressful’:

"In state, you’ve got three levels of people below you, so if you’re … a state consultant, yes you’ve got different stresses, you’ve got to give a lecture and you’ve got to give that, but I’m saying that’s a different type of stress. But on a clinical responsibility level, between you and the patient, there’s an intern and a registrar… So the family’s complaining … and that comes all the way through those two people before it gets to you. So that’s like you’re three degrees removed. (H1 DP18, Interview 1)"

In summary, the public sector seems to generally offer specialists social satisfaction through collegial interaction and learning, but less satisfaction with relationships with other hospital and health workers, particularly nurses, compared with the private sector. The public sector also has the potential for specialists to feel more supported through the greater availability of managers, as was the case in H1, though this may not be so in other hospitals. Distrust of the public hospital ‘administration’ and DoH, meanwhile, seemed universally high. Patient relationships also seem to be strained in the public sector, due to relative unwillingness or inability of patients to follow directions, as well as, potentially, some classism and racism among doctors. On the other hand, private patients are often seen as highly demanding. The amount of doctor-patient interaction in the private sector may also be overwhelming to some specialists.

### The work itself

Public sector work was noted as highly intense by respondents, with more serious pathology, since patients generally come from poorer backgrounds and present later. Some doctors mentioned thriving on the ‘challenge’ of such work, and that there was nothing worse than ‘a simple list of tonsils’. Others seemingly preferred more ‘straightforward’ work, which was more common in the private sector. One respondent noted, meanwhile, that ‘when learning you want to see the worst’, but that you can get ‘tired of it’ quickly. Personal values for particular modes of work seemed critical determinants of satisfaction, in these respects:

"… some people are more suited to private… there are people who want to go and do the less complicated stuff, and they enjoy… [working] on that level. And for them to do the more complicated stuff that would be a nightmare. So … everyone makes their own choices I suppose… (H1 DP2, Interview 1)"

Specialists also generally enjoyed the research and teaching aspects of public academic work, in part because it added variety to their job. One respondent said of the public sector, ‘I’m never going to have a boring, routine day’, while another noted:

"…it is good and interesting to have students around you. So the teaching component of it I’ve always found just varies your day. It adds a little bit of an extra dynamic to what your routines are, so it can be quite fun and it’s… a little bit challenging, and it just… adds spice to all your humdrum things. (H1 DP7, Interview 1)"

Interestingly, satisfaction with public work may also come down partly to the need to feel needed. Public doctors generally seemed to feel more important and needed, if not always fully appreciated, by their patients; as opposed to feeling less ‘relevant’ in the private sector or abroad, as one doctor among many:

"…I felt I was making a difference [in public]. So [in] the state sector… you feel like that all the time, and the operational constraints are such that you always feel that you’re needed there… I’ve worked in other environments, overseas and where you felt you were a small cog in a big machine and that no one was really going to notice if you don’t come to work because you’re easily replaced. You don’t have that [in H1], you feel like you’re making a tangible difference to people’s lives. (H1 DP10, Interview 2)"

There thus seemed to be a number of advantages to public sector work for specialists. A private-based specialist lamented he missed public work, in fact, and had only left the public sector because he felt his financial needs were unmet:

"…I think solo [private] practice on its own, full-time, is a lonely, quite a limiting type of existence. I think you become very sort of limited in what you do, you tend to see similar problems most of the time. (H1 DP)"

There were exceptions, of doctors who performed very specialized, elective private work that was not possible in the public sector, but these seemed to be exceptions rather than the rule. In addition, ‘challenging’ work, for those who valued it, seemed particularly hard to come by for those just establishing a private practice, who were noted to have to take whatever work they could get. One H1 respondent explained he felt the public sector had a lot to offer in terms of satisfaction with the work itself, and specialists elsewhere often deluded themselves in this respect:

"I mean it’s often interesting to listen to people’s justifications for why they leave. I mean when people leave they have to have good reasons, and so they often make those reasons [up]; and it’s the same with people who emigrate. They often don’t like coming back, because it’s actually not as bad as the reasons they describe of why they left, and often I think the guys in private think that that’s the land of milk and honey, that there’s fantastic medicine going on and they’re doing fantastically interesting cases… [but] there are general surgeons out there whose whole career revolves around circumcisions and hernia repairs. And they’re specialist surgeons. And there’s no specialist surgeon in H1 doing those kinds of things, it’s only the junior surgeons… They make a good living out there, but it’s not interesting. And they get very good at doing circumcisions. (H1 DP2, Interview 2)"

The ability to work with more autonomy in the private sector, however, did appear to carry a distinct advantage for those who valued it. This seemed particularly true of those frustrated with public ‘regulations and rules’, who wanted to work on their own terms:

"Okay, I enjoy the job [in H1], but there are lots and lots and lots of frustrations here. And as I say, most of the frustration is this whole thing [attitude] of ‘can’t do’ and regulations and rules, and pettiness, and there’s a lot of politics around here. Everybody’s more important than everybody else. Out there [in private] … you do your job, you come and go, you organize your practice as you want to. You know if I want to put three flowers on my desk, then I put them on the desk; if I want them on the floor I put them on the floor. It’s my choice, and if it keeps me and my patients happy, nobody worries about it. (H1 DP1, Interview 1)"

As such, the work itself seemed generally preferred in the public sector for the cohort of dual practice specialists interviewed, since academic medicine, particularly, presented opportunities for more ‘interesting’, ‘complex’ pathology, as well as for research and teaching. This preference may have been exaggerated by sampling, since respondents were overwhelmingly working at least part-time in the public academic sector, and so may intrinsically value this work more.

#### Summary of results

The above determinants of job satisfaction in the public *versus* private sector are summarized in the table below. Satisfiers, rather than dissatisfiers, are presented, given that dissatisfiers in one sector are essentially satisfiers in another, since specialists interviewed were commenting on what was (dis)satisfying about one sector relative to the other. Thus dissatisfiers are excluded to avoid duplication of the same factors. For example, being more dissatisfied with resource availability in the public sector is presented as being satisfied with resource availability in the private sector (Table 1).

**Table 1:** **Summary of different job satisfiers in public *vs***. **private sectors, for specialists**

**Category**	**Public satisfiers**	**Private satisfiers**
Rewards	+ Good benefits (for example, paid sabbatical leave, state pension), stable income	+++ Much better pay generally (depending on level of specialization)
Physical environment	++ More predictable working hours - less ‘selling availability’	
		+++ Higher resource availability (incl. vis. other health workers)
		++ Greater sense of career path or progression through more prestige and recognition
Social environment	+++ More collegial relations among doctors	
	+ Good relations with managers (in H1, not necessarily elsewhere)	+++ Fewer stresses with ‘the administration’ and National/provincial DoH through self-employment
	++ Patients less demanding, and less patient contact	++ Patients viewed as more compliant, more patient contact
		+++ More positive social relations with other health and hospital workers
Work itself	+++ Opportunities for research and teaching (in academic settings)	
	+++ More opportunities for ‘challenge’, variety, and feeling ‘needed’ or relevant	+ More ‘straightforward’ and less complicated cases (valued by some)
		+ Opportunity for state-of-the-art medical practice experience
		+++ More autonomy and ability to influence working environment

Source: qualitative evidence.

Note: ‘+’ signs signify estimates/guesses from the author of how important each job aspect appeared to respondents in the qualitative case study, with a score between 1(+) and 3(+++). Higher scores were accorded based around popularity of the issues and how clear and strong arguments were in their favor.

The table points to a number of advantages to private, relative to public, sector jobs, including: more money, in general; the ability to work in a relatively resource-rich environment; more of a sense of career progression; fewer stresses with ‘the administration’ (upper management) and DoH; better social interactions with nurses and other health workers; and more working autonomy. This list gives important detail on why specialists are leaving the public sector for the private sector in South Africa.

The table also highlights certain advantages to public sector work, including: more predictable working hours and job security; a more collegial social work environment; greater ability to do work that feels more ‘needed’ and ‘challenging’; and in academic hospitals, having the ability to undertake research and teaching work. This list is equally important as that above, as it shows why some specialists choose not to migrate from the public sector.

## Discussion

The results above enhance understanding of both reasons for specialists migrating to the private sector, and reasons for some staying in the public sector. This summary may well represent the first relatively complete list of factors that are driving South African specialists into the private sector, as well as keeping some from leaving.

The results must be interpreted carefully, however, since the dual practice doctors interviewed are likely to value aspects of both public and private sector work, having self-selected into multiple (public-private) job arrangements that allow them to work in both. Other specialists in South Africa may value public *versus* private work differently; for instance, private-only specialists will likely value private incentives more, such as higher wages and more working autonomy, while collegial interactions and ‘challenging’ pathology may be less valued by them.

The emphasis placed on different job satisfaction factors above is also based on a specific, urban context in one public and one private hospital; and specialists in different hospitals may place different emphasis on the job satisfaction factors outlined above. For example, in hospital contexts where academic (teaching and research) work is less available than in H1, this may not play a strong role in job satisfaction.

Previous studies of doctors’ motivations in South Africa tend to focus more on doctors who have not specialized, and on the rural context ( [39-41], and also see [53-55]). This also highlights the context-specificity of this article’s research, although the overall list of job satisfaction factors above has significant cross-overs with the aforementioned studies. Yet some differences are inevitable. In particular, this article focuses more on incentives relating to research, academic work, and overseeing large teams in the public sector, factors which are less available to those who are less specialized and/or working in rural areas.

The results point towards a number of policy implications for urban medical specialists, at the very least, that are worthy of further discussion. The most important of these, perhaps, is that it may not be additional income that is primarily needed, or which may be most cost-effective, to increase job satisfaction and retention for those currently working in the public sector. This is since, although public specialists often appear to earn significantly less than they could by working full-time in the private sector, they generally singled out other factors as mostly lying behind their frustrations. This might be due, in part, to salary increases that public sector specialists received prior to the study, as part of doctors’ ‘Occupation-Specific Dispensation’ packages; but it is also no doubt due in part to different income expectations among public-based specialists.

This finding is consistent with results from Pillay in 2009 [22], whose job satisfaction survey of nurses in South Africa indicated that it was work context factors (including resource availability), rather than pay-related factors, that most underscored the difference between public and private job satisfaction. The motivational human resources for health literature has also argued that increasing wages to levels comparable to the private or overseas sector may be less affordable than addressing non-financial incentives in relatively resource poor contexts [34,56,57]. These ideas are also broadly consistent with Maslow’s theory that after a certain threshold, wages become relatively less imperative a factor in determining job satisfaction.

The implications are that the South African National Department of Health may well need to prioritize improving non-financial incentives before considering financial ones further to improve retention. Resource, including staff, availability should be addressed as a priority, since this seems a particularly salient determinant of job dissatisfaction in the public, relative to private, sector. This may be achieved through investing in equipment and machinery and the like, or through adequate equipment maintenance.

Another option is to increase support staff availability, that is, employ more cleaners, porters, and hospital administrators. This could represent something of an ‘easy win’ for the DoH, given support staff wages are relatively inexpensive, yet these workers play an important role in making specialists more efficient and satisfied – by reducing their time spent on lower skilled tasks. This highlights the need to address the efficiency of staff mix in South African hospitals, which implies current resources could be more efficiently and better utilized, potentially without the need to spend more money.

Other interventions to improve public sector retention may include re-evaluating the career ladder and wage structure of public doctors, in order to promote a clearer sense of ongoing career development, particularly at the senior specialist level. More fundamental, perhaps, is the need to address the distrust that public specialists exhibit for the DoH, hospital and provincial upper management. Most obviously, the DoH could work to ensure health workers and specialists are adequately consulted on policy changes that affect them, and that these consultations are advertised and publicized, which may be equally important. Inclusive policy-making, in this regard, may be perceived as more fair, and less ‘imposed’ [58]. While this may not always be practical or realistic, the DoH should at least place more effort in communicating a clear vision or strategy for the health sector, and on what basis they are making their decisions.

Again, this implies that it does not necessarily cost much more money to better retain public specialists. Rather, as Penn Kekana *et al*. [59] highlight in their ethnography of maternal health services in South Africa, the dialogue that goes into health system policy change may need to be reconsidered, with more focus on holistic understanding of all tiers at which policy changes risk alienating front-line health workers, and less focus on vertical interventions that do not take the wider health system context into account.

Finally, it may be important not just to address particular weak points in job satisfaction in the public sector, but also to emphasize and capitalize on areas in which satisfaction is relatively strong. Since the public sector is often valued for its collegial atmosphere and the availability of academic work, for instance, it may be possible to attract more private specialists back to the public sector, on a part-time basis, to undertake this work. This has been planned under a model of GP and specialist contracting into the private sector under NHI [12]. This may be important to build upon going forward since, like training more health workers, it has the potential to make a marked difference by taking the burden off those who currently choose to remain in the public sector where they are greatly needed.

## Competing interests

The author declares that he has no competing interests.
